# Adrenal sensitivity to stress is maintained despite variation in baseline glucocorticoids in moulting seals

**DOI:** 10.1093/conphys/cov004

**Published:** 2015-03-11

**Authors:** Cory Champagne, Michael Tift, Dorian Houser, Daniel Crocker

**Affiliations:** 1 National Marine Mammal Foundation, San Diego, CA 92106 , USA; 2 Scripps Institution of Oceanography, La Jolla, CA 92093 , USA; 3 Sonoma State University, Rohnert Park, CA 94928 , USA

**Keywords:** Stress, marine mammal, hypothalamic–pituitary–adrenal axis, aldosterone

## Abstract

Stressful disturbances activate the hypothalamic–pituitary–adrenal (HPA) axis and result in the release of glucocorticoid (GC) hormones. This characteristic stress response supports immediate energetic demands and subsequent recovery from disturbance. Increased baseline GC concentrations may indicate chronic stress and can impair HPA axis function during exposure to additional stressors. Levels of GCs, however, vary seasonally and with life-history stage, potentially confounding their interpretation. Our objective was to evaluate HPA axis function across variations in baseline GC levels. Northern elephant seals show substantial baseline variation in GC levels during their annual moulting period. We therefore conducted measurements early, in the middle and at the end of moulting; we simulated an acute stressor by administering adrenocorticotrophic hormone and evaluated the changes in circulating hormones and metabolites over the following 2 h. The stress response was characterized by increases in both cortisol and aldosterone (*F*_7,105_ = 153 and 25.3, respectively; *P* < 0.001). These hormones increased in parallel and the slopes of their relationship varied by study group, suggesting they are regulated in a co-­ordinated manner during acute stress in this species. There was no detectable difference in the total release of cortisol or aldosterone among study groups, indicating that the HPA axis remained sensitive to stimulation by adrenocorticotrophic hormone despite varying baseline levels of GCs. Acute stress influenced carbohydrate and fat metabolism in all study groups, but protein catabolism was affected to a far lesser degree. These findings suggest that elephant seals, and potentially other pinniped species, are resilient to moderate variations in baseline GC levels and remain capable of mounting a response to additional stressors.

## Abstract (precis)

Select hormones, like glucocorticoids, could be informative markers of stress in animals. To be useful, however, baseline and stressed state hormone concentrations must be described. We therefore evaluated the timing and magnitude of stress hormone release by simulating an acute stressor in a marine mammal, the northern elephant seal.

## Introduction

**Figure 1:**
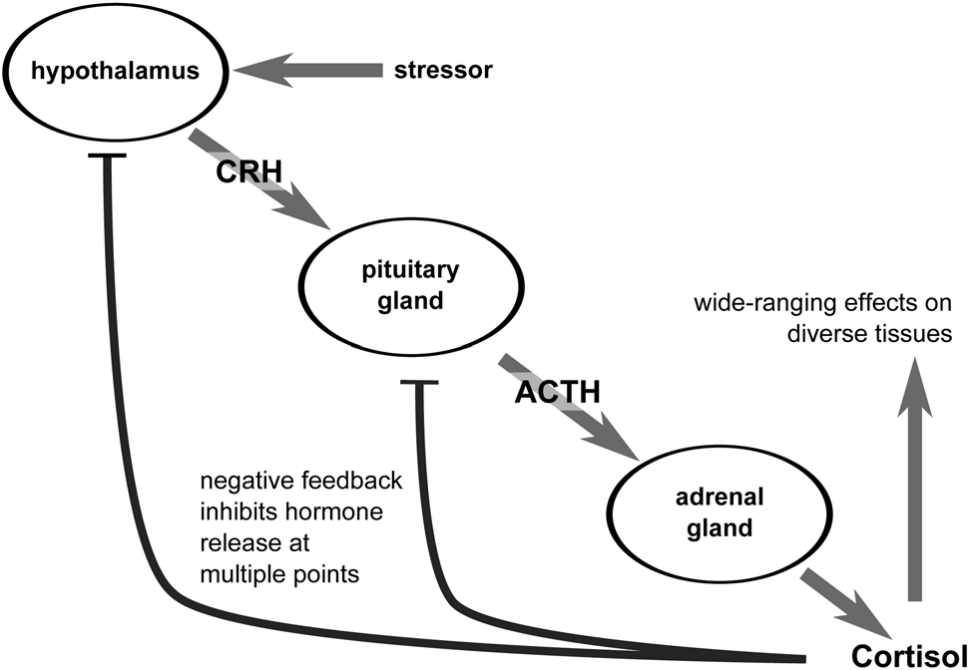
The hypothalamic–pituitary–adrenal axis is activated during stress. Glucocorticoid hormones (e.g. cortisol) are released and influence diverse target tissues. Abbreviations: ACTH, adrenocorticotrophic hormone; CRH, corticotrophin-releasing hormone.

Numerous environmental conditions potentially disrupt homeostasis in free-ranging animals. Sufficient disturbance will result in a stress response, characterized by activation of the hypothalamic–pituitary–adrenal (HPA) axis and the release of glucocorticoid (GC) hormones (e.g. cortisol; Fig. 1). Activation of the HPA axis and the release of GCs have wide-ranging consequences and influence several metabolic pathways; for example, at high concentrations GCs increase gluconeogenesis, lipolysis and protein catabolism (Sapolsky *et al.*, 2000). These adjustments support immediate energetic demands at the expense of energy reserves. Many stressors result from or are influenced by human activity (Maxwell *et al.*, 2013). Conservation biologists are therefore increasingly using stress assessments to monitor animal health and inform management efforts (Cooke and O'Connor, 2010; Madliger and Love, 2014), but this requires a far more detailed understanding of stress responses in wildlife systems than is currently available (Dantzer *et al.*, 2014).

Baseline levels of GCs provide insight into the stress state of individuals and are frequently measured in free-ranging animals. These levels, however, vary widely among species and with life-history stage (Romero, 2002). The interpretation of these metrics is further complicated because increased baseline GCs may be associated with increased, decreased or unrelated to metrics of fitness, including survival (Busch and Hayward, 2009). This probably results from the concentration-­dependent function of GC hormones. At low levels, GCs interact with high-affinity type I receptors and influence metabolic functions; it is not until GC concentrations increase markedly that they bind with low-affinity type II receptors and stimulate a stress response (Romero, 2004). Even GC levels that are chronically increased during predictable life-history events are not necessarily deleterious to the individual (Boonstra, 2013). They may, however, still influence an animal's capacity to respond to additional stressors. The ability of an individual to mount a response, measured as the magnitude of GC release following a stressor, may provide a better metric of animal fitness (Romero, 2004).

As a result of their amphibious lifestyle, pinnipeds (seals and sea lions) are exposed to a multitude of aquatic and terrestrial stressors during critical life-history stages. For example, many pinnipeds endure prolonged periods of food deprivation simultaneous with energetically costly activities, including breeding, lactation and moulting (Houser *et al.*, 2013). These periods occur at predictable intervals and are associated with hormonal changes that potentially influence the ability to respond to additional stressors (Costa, 1993; Atkinson *et al.*, 2011). For example, circulating cortisol concentrations frequently increase during fasting in pinnipeds (Ortiz *et al.*, 2001; Guinet *et al.*, 2004). Several pinniped populations are in decline (e.g. Steller sea lions, *Eumetopias jubatus*), while others are stable or increasing (e.g. California sea lions, *Zalophus californianus*, and northern elephant seals, *Mirounga angustirostris*; Atkinson *et al.*, 2008). Understanding how normal variation in baseline GC levels influences the capacity of animals to respond to subsequent stressors provides insight into how cumulative stressors may influence the resilience of animals to environmental disturbance and its potential fitness consequences.

Our objective was to evaluate the influence of natural variation in baseline GC concentrations on the HPA axis and its sensitivity to additional stressors. Periods when GC levels are elevated to support life-history functions may limit the ability of animals to respond adequately to additional stressors (Rich and Romero, 2005). For example, GC levels increase during moulting in several pinniped species (Riviere *et al.*, 1977; Ashwell-Erickson *et al.*, 1986; Boily, 1996; Myers *et al.*, 2010; Gobush *et al.*, 2014). Elephant seals are an ideal species in which to investigate the stress response in a free-ranging marine mammal because a considerable amount of previous work has investigated their metabolism and its regulation. Elephant seals are known to have considerable variation in GCs as a function of age, sex and life-history stage (including breeding and moulting; for review see Houser *et al.*, 2013). To investigate the possibility that that increased GC concentrations influence the stress response, we examined the response to a simulated stressor—the administration of exogenous adrenocorticotrophic hormone (exACTH)—across the annual moult of juvenile seals when GC levels are anticipated to vary.

We tested the hypothesis that the magnitude of the stress response would vary during moulting and predicted that increases in baseline GCs would result in a muted response to a simulated stressor. In this species, typical handling procedures using dissociative anaesthetics do not appear to cause a significant stress response, evidenced by stable cortisol concentrations during handling, thus offering a study system amenable to experimental perturbation with minimal handling artifact (Champagne *et al.*, 2012b). To assess the stress response, we quantified several hormones and metabolites. Cortisol and endogenous ACTH were used as metrics of the HPA axis response. Some studies have found that ACTH stimulates aldosterone secretion in marine mammals (St Aubin and Geraci, 1986; Gulland *et al.*, 1999; Ensminger *et al.*, 2014); we therefore also evaluated circulating aldosterone as a potential stress hormone. The thyroid hormone axis may be influenced during stress, notably by promoting the production of reverse triiodothyronine (rT_3_; Weissman, 1990); thus, rT_3_ was measured to assess the influence of acute stress on the thyroid axis. The stress response has further downstream consequences on metabolism, so select metabolites were assessed to evaluate metabolic effects. We measured blood urea ­nitrogen (BUN), non-esterified fatty acid (NEFA), glucose and lactate concentrations as metrics of protein catabolism, lipolysis and carbohydrate metabolism, respectively.

## Materials and methods

This study was conducted at Año Nuevo state park, San Mateo County, CA, USA. Each spring, juvenile elephant seals haul out on rookeries and undertake a catastrophic moult, completely replacing their pelage. The moulting process takes ~1 month, during which elephant seals fast completely from food and water; simultaneously, hormone changes occur, including changes in GCs, to facilitate moulting (Champagne *et al.*, 2012a; Houser *et al.*, 2013). Measurements were conducted in 16 yearling northern elephant seals (seven female and nine male) at the beginning (*n* = 6), middle (*n* = 5) and end (*n* = 5) of the spring moulting period (early, mid, and late, respectively) in a cross-sectional sampling design. Animal states were determined based on the degree of moulted pelage. Based on pilot studies, these stages showed substantial variability in baseline GC concentrations, and the predicted trends were observed in the present study (see Results and Table 1).

**Table 1:** Sex, initial mass, hormone and metabolite concentrations for each of the three study groups

Study group	F/M	Mass (kg)	ACTH (pm)	Cortisol (nm)	Aldosterone (pm)	Glucose (mm)	Lactate (mm)	NEFA (mm)	BUN (mm)	rT_3_ (nm)
Early	2/4	155 (15)^a^	4.08 (1.53)	110 (38)^a^	233 (77)^a^	6.80 (0.69)^a^	3.58 (0.77)^a^	0.864 (0.168)	7.43 (0.73)^ab^	1.47 (0.31)^a^
Mid	3/2	133 (11)^b^	3.83 (0.87)	413 (111)^b^	1431 (1056)^b^	5.52 (1.01)^b^	5.17 (0.83)^b^	0.906 (0.041)	9.56 (1.82)^a^	3.31 (0.59)^b^
Late	2/3	121 (9)^b^	2.86 (0.83)	306 (129)^b^	372 (185)^a^	6.97 (0.55)^a^	3.02 (1.14)^a^	1.210 (0.372)	6.18 (1.81)	2.46 (0.17)^c^
*P*-Value	NA	<0.01	nsd	<0.001	<0.001	<0.05	<0.01	nsd	<0.05	<0.05

Results are expressed as mean values (SD). Significant differences were evaluated by ANOVA. *P*-Values are shown in the bottom row; *post hoc* pairwise comparisons were performed using Tukey's HSD test, and differences between study groups are denoted by differing letters within each column. Abbreviations: ACTH, ­adrenocorticotrophic hormone; BUN, blood urea nitrogen; F, female; M, male; NA, not applicable; NEFA, non-esterified fatty acid; nsd, no significant difference; rT_3_, reverse triiodothyronine.

Study animals were chemically immobilized as previously described (Kelso *et al.*, 2012). Briefly, sedation was induced with 1 mg/kg tiletamine–zolazepam (Telazol) administered as an intramuscular injection and maintained using periodic intravenous doses of ketamine and diazepam (all drugs from Fort Dodge Laboratories, Fort Dodge, IA, USA). This sedation technique does not elicit a stress response in this species because circulating cortisol concentrations do not increase markedly during sedation (Champagne *et al.*, 2012b). Blood samples were collected via an 18 gauge, 8 cm needle inserted into the extradural vessel. Initial blood samples were collected immediately after study animals were sedated, in order to establish baseline hormonal and metabolite values. An intramuscular dose of 30 U exACTH (Wedgewood Pharmacy, Swedesboro, NJ, USA) was then administered via an 18 gauge 8 cm needle into the lateral musculature (0.22 U/kg, SD 0.007 U/kg). Blood samples were then collected periodically for 2.5 h after exACTH injection (15, 30, 60, 90, 120 and 150 min after administration). At the ­conclusion of the measurement, seals were weighed using a scale suspended from a tripod (MSI tension dynamometer, Seattle, WA, USA).

The response to exACTH administration was evaluated by quantifying select hormones and metabolites. Hormone responses were measured in serial samples for cortisol, aldosterone and endogenous ACTH, whereas rT_3_ was measured in only the initial and final samples (pre and post, respectively). Hormones were assayed using commercially available radioimmunoassay (RIA; cortisol and aldosterone from Siemens, Inc., Washington, DC, USA; and rT_3_ from Alpco, Inc., Salem, NH, USA) or enzyme immunoassay (EIA) kits (ACTH from Alpco, Inc.). All kits have been previously validated for northern elephant seals (cortisol, Ortiz *et al.*, 2001; aldosterone, Ortiz *et al.*, 2000; Houser *et al.*, 2001; and ACTH and rT_3_, Ensminger *et al.*, 2014). The responses of glucose and lactate were measured in serial samples using a dedicated autoanalyser (YSI, 2300; Yellow Springs Inc., Yellow Springs, OH, USA). Non-esterified fatty acid and BUN concentrations were ­measured from initial and final samples (pre and post, respectively), using enzymatic colorimetric assays (Wako Diagnostics, Richmond, VA, USA; and Stanbio, Boerne, TX, USA, ­respectively).

**Figure 2:**
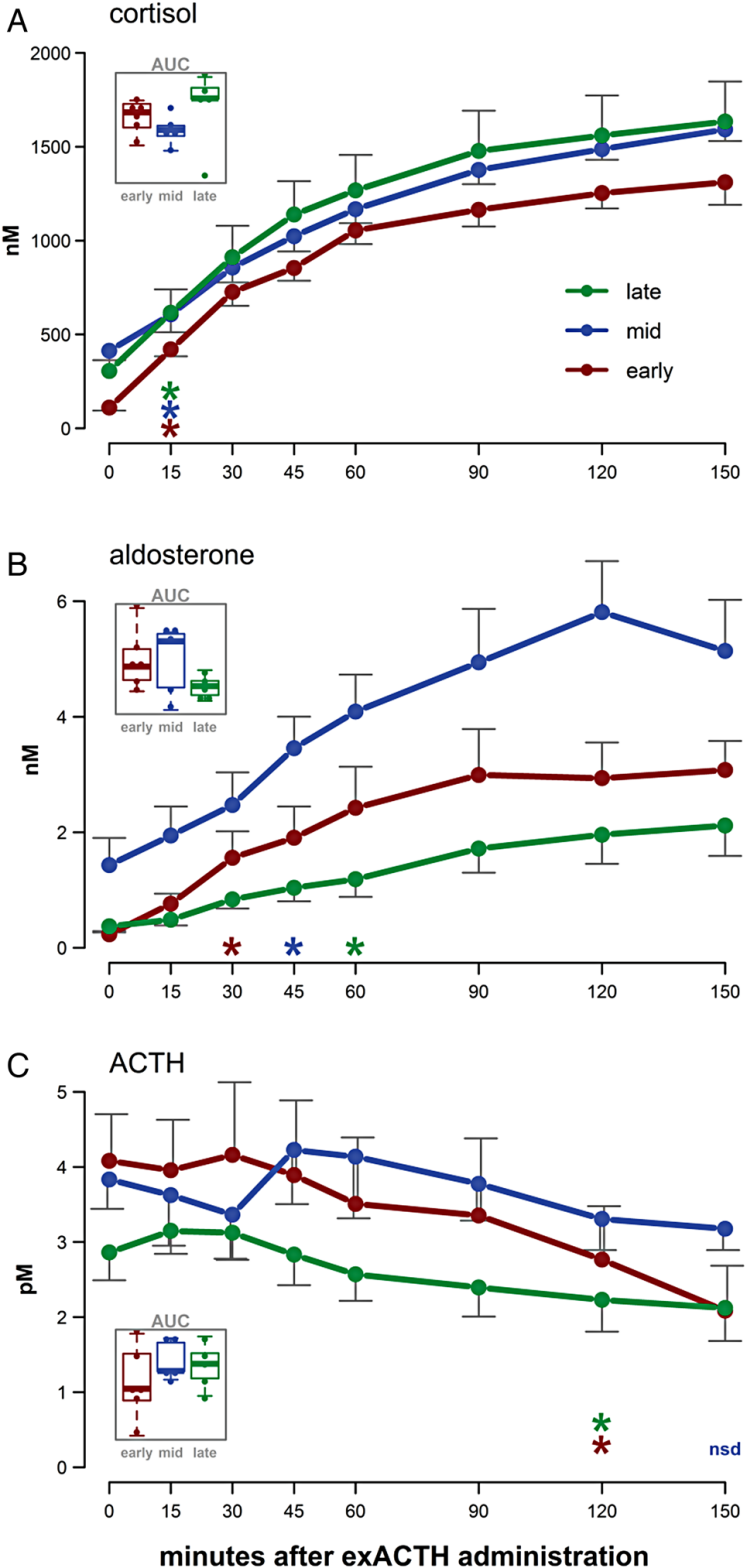
The responses of cortisol (**A**), aldosterone (**B**) and endogenous adrenocorticotrophic hormone (ACTH; **C**) to the administration of exogenous ACTH (exACTH) for each of the three study states: early, middle and late moulting. Initial values are shown at time zero. Coloured asterisks indicate that the hormone value at that time point and all subsequent time points differs significantly from the initial hormone value (Dunnett's test, *P* < 0.05); ‘nsd’ indicates no significant difference from time zero for any sample in that group. Error bars represent the SEM at each sampling time. An inset within each panel shows a comparison of the total hormone release over time, as area under the curve (AUC) values, among study groups; no differences in AUC values were detected, and the *y*-axis units are concentration × minutes (not shown).

To assess the total response during the experimental period, we calculated the total area under the curve (AUC) over time by summing the areas under the response vs. time polygons between sampling points relative to their initial concentrations (see Fig. 2). Statistical tests were performed in R, version 3.0.2 (R Core Team, 2013). Response variables were logarithmically transformed when necessary to meet distribution and variance assumptions. Differences among groups were assessed using ANOVA or Welch's one-way test assuming unequal variances, and *post hoc* pairwise comparisons were conducted using Tukey's tests. Differences among repeated samples were evaluated using linear mixed models (LMMs), with individual as a random effect and sample time as an ordered fixed effect. Appropriate degrees of freedom within LMMs were estimated using the Kenward–Rogers approximation and *P*-values calculated in the lmerTest package (Kuznetsova *et al.*, 2014); *post hoc* comparisons among repeated samples were conducted using Dunnett's test against the initial (time zero) sample within each group. Goodness of fits were calculated using the MuMIn package (Kamil, 2014) based on the methods of Nakagawa and Schielzeth (2013) to determine *r*^2^ metrics within LMMs. Marginal *r*^2^ (m*r*^2^) values are reported to describe the variance explained by fixed effects.

## Results

Cortisol and aldosterone showed similar overall trends in their baseline concentrations; cortisol was lowest early in moulting (*F*_2,13_ = 13.7, *P* < 0.001; Table 1) and aldosterone was greatest mid-moult (log_10_ transformed, *F*_2,13_ = 14.7, *P* < 0.001; Table 1). There was no detectable difference among initial ACTH concentrations (*P* > 0.1). Glucose and lactate had opposing trends across moulting; glucose concentrations were lowest in the middle of moulting when lactate concentrations were highest (*F*_2,13_ = 5.5, 7.5, respectively; *P* < 0.05; Table 1). There was no detectable difference in NEFA concentrations among study groups (*P* > 0.1; Table 1). Blood urea nitrogen concentrations were lowest at the end of moulting (*F*_2,13_ = 6.5, p = 0.01; Table 1). There was considerable variability in rT_3_ concentrations, and each study group was different from the others (*F*_2,13_ = 30.2, *P* < 0.0001; Table 1).

**Figure 3:**
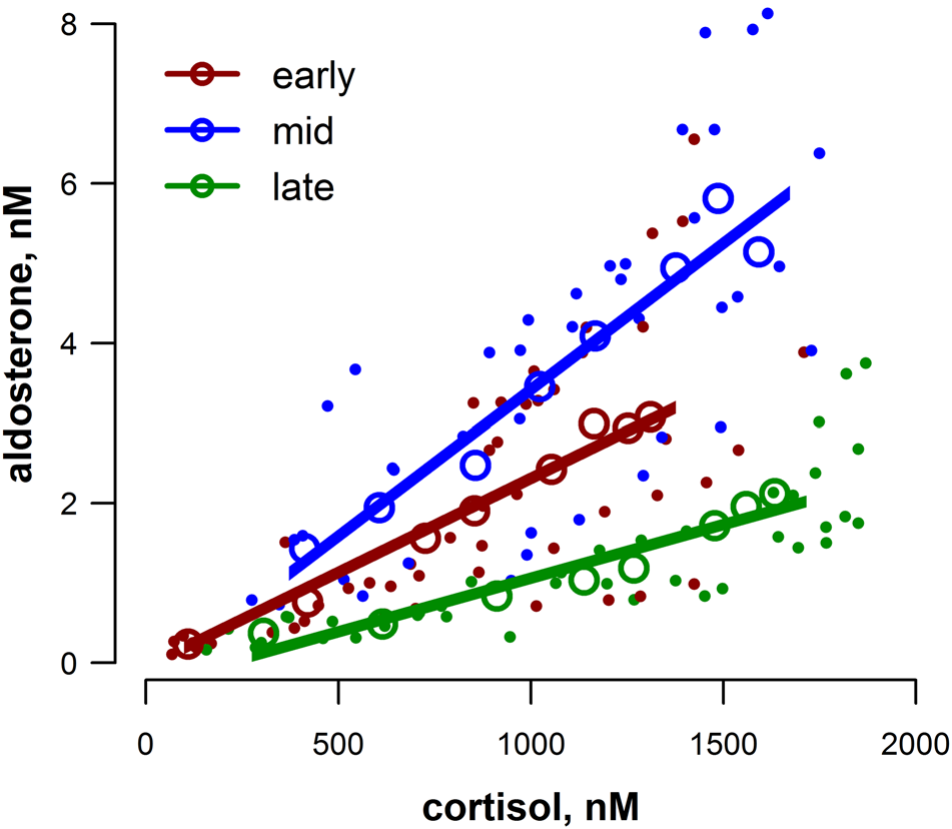
Cortisol and aldosterone concentrations were tightly correlated during the stress response [linear mixed model (LMM), *F*_1,113_ = 237.5, *P* < 0.0001], and this relationship varied by study group (cortisol × state, *F*_2,113_ = 17.1, *P* < 0.0001); see main text for further description of statistical analysis. Each sample collected in this study for all study animals is represented by filled symbols and colour coded according to moulting state (early, mid or late). Fitted regression lines are derived from the LMM model fits, and marginal *r*^2^ (m*r*^2^) values are shown for each group. Open circles are average hormone values at each sampling time (0–150 min) within the study group and are shown for visual clarity only.

The timing and magnitude of cortisol, aldosterone and ACTH responses following exACTH administration are shown in Figure 2. Concentrations of both cortisol and aldosterone were significantly elevated following ACTH administration (cortisol, *F*_7,105_ = 153, *P* < 0.001; and log_10_(aldosterone), *F*_7,105_ = 25.3, *P* < 0.001). In all three study groups, cortisol concentration increased within 15 min and remained elevated for the duration of the experiment (Dunnett's comparison against the initial sample within each group, *P* < 0.05). There was an increase in the response time of aldosterone following exACTH administration with the progression of moulting; aldosterone was significantly increased within 30 min early in moulting, 45 min in the middle of moulting, and 60 min in the late moulting group (Dunnett's comparison against the initial sample within each group, *P* < 0.05). We did not detect a difference in the total release of cortisol or aldosterone among the study groups, as measured by AUC relative to initial concentrations (ANOVA, *P* > 0.1: Fig. 2A and B insets). To assess the relationship between cortisol and aldosterone responses, we ran a linear mixed model including moulting state as a factor and individual as a random effect; cortisol and aldosterone increased in parallel following exACTH administration (*F*_1,113_ = 237.5, *P* < 0.0001; Fig. 3), and the slopes of the relationship varied by study group (cortisol × state, *F*_2,113_ = 17.1, *P* < 0.0001).

There was a significant decrease in ACTH following exACTH administration (*F*_7,105_ = 9.2, *P* < 0.001). This decline, however, did not occur until at least 120 min after exACTH administration (Dunnett's comparison against the initial sample within each group, *P* < 0.05; Fig. 2C).

**Figure 4:**
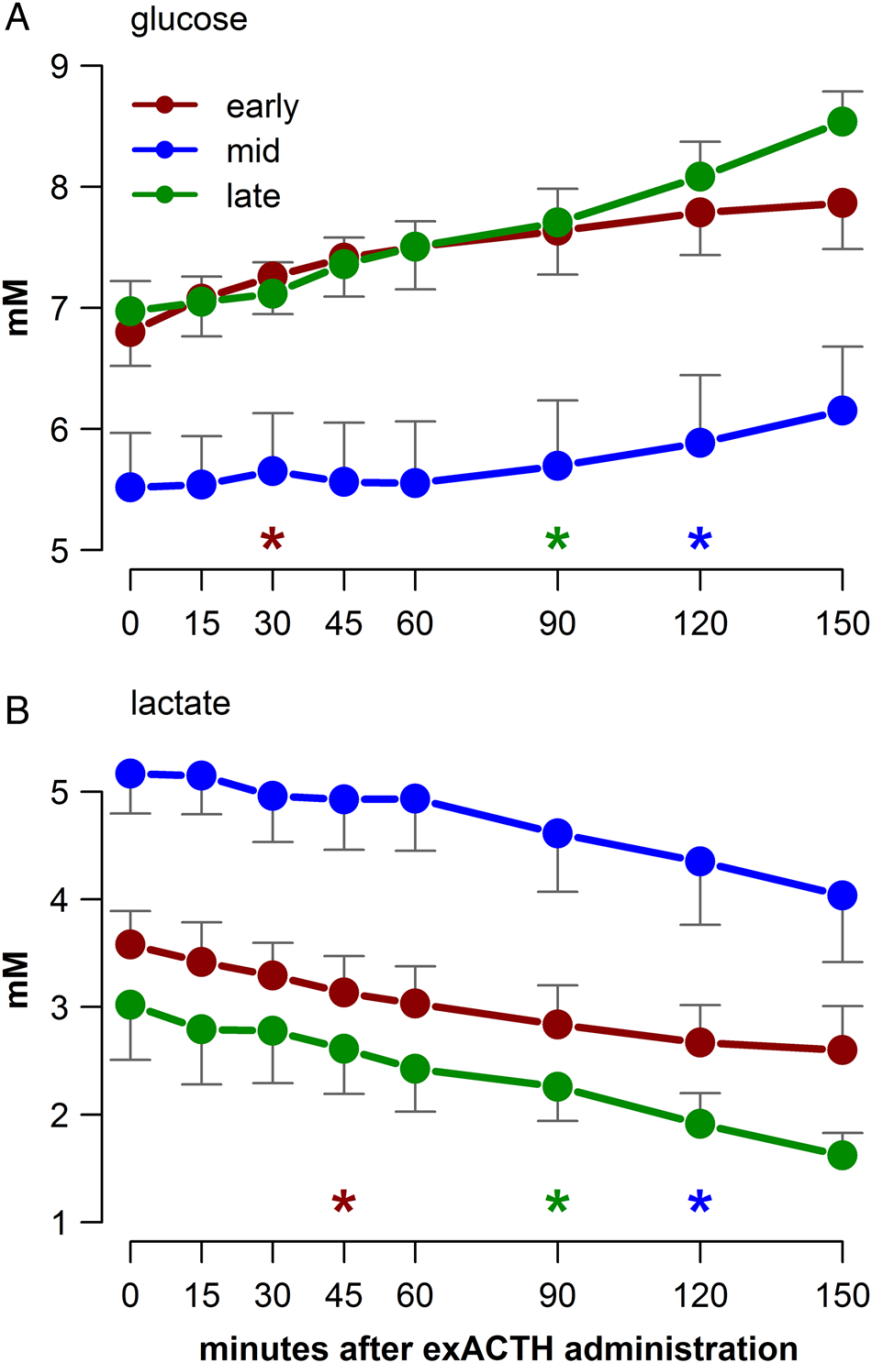
The responses of glucose (**A**) and lactate (**B**) to exACTH administration for each of the three study states: early, middle and late moulting. Initial values are shown at time zero. Coloured asterisks indicate that the hormone value at that time point and all subsequent time points differs significantly from the initial hormone value (Dunnett's test, *P* < 0.05). Error bars represent SEM at each sampling time.

**Figure 5:**
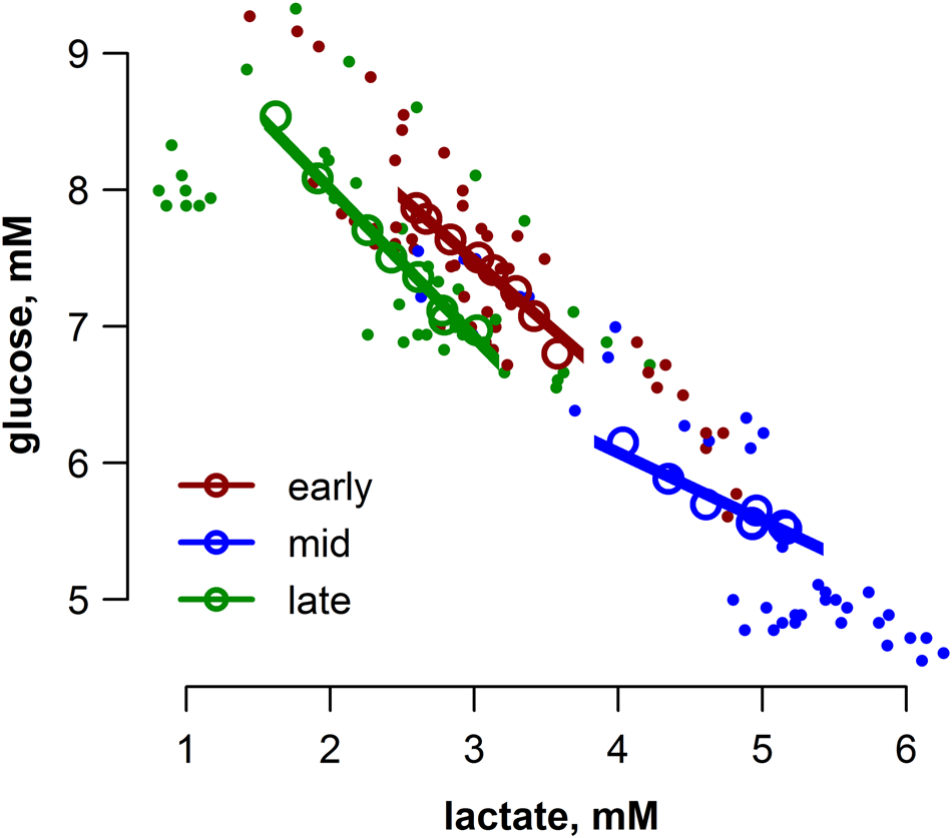
Glucose and lactate concentrations were inversely related during the stress response (LMM, *F*_1,113_ = 905.1, *P* < 0.0001), and this relationship varied by study group (lactate × state, *F*_2,113_ = 42.0, *P* < 0.0001); see main text for further description of statistical analysis. Each sample collected in this study for all study animals is represented by filled symbols and colour coded according to moulting state (early, mid or late). Fitted regression lines are derived from the LMM model fits, and marginal *r*^2^ (m*r*^2^) values are shown for each group. Open circles are average values at each sampling time (0–150 min) within the study group and are shown for visual clarity only.

Circulating concentrations of glucose and lactate displayed opposing responses to exACTH administration; glucose increased (*F*_7,105_ = 20.6, *P* < 0.001; Fig. 4A), while lactate decreased (*F*_7,105_ = 25.4, *P* < 0.001; Fig. 4B) over time in all study groups. There were no detectable differences in the total glucose or total lactate responses among study groups as measured by AUC (ANOVA, *P* > 0.1). To assess the association between glucose and lactate, we ran a linear mixed model with moulting state as a cofactor and individual as a random effect; glucose and lactate were inversely related (*F*_1,113_ = 905.1, *P* < 0.0001; Fig. 5), and the slopes of the relationship varied by study group (lactate × state, *F*_2,113_ = 42, *P* < 0.0001). Furthermore, to assess the total responses of ­glucose and lactate, we ran a general linear model between glucose and lactate AUC values, with moulting state as a cofactor. The total responses of glucose and lactate were inversely related (*F*_1,10_ = 80.2, *P* < 0.0001; Fig. 5).

**Figure 6:**
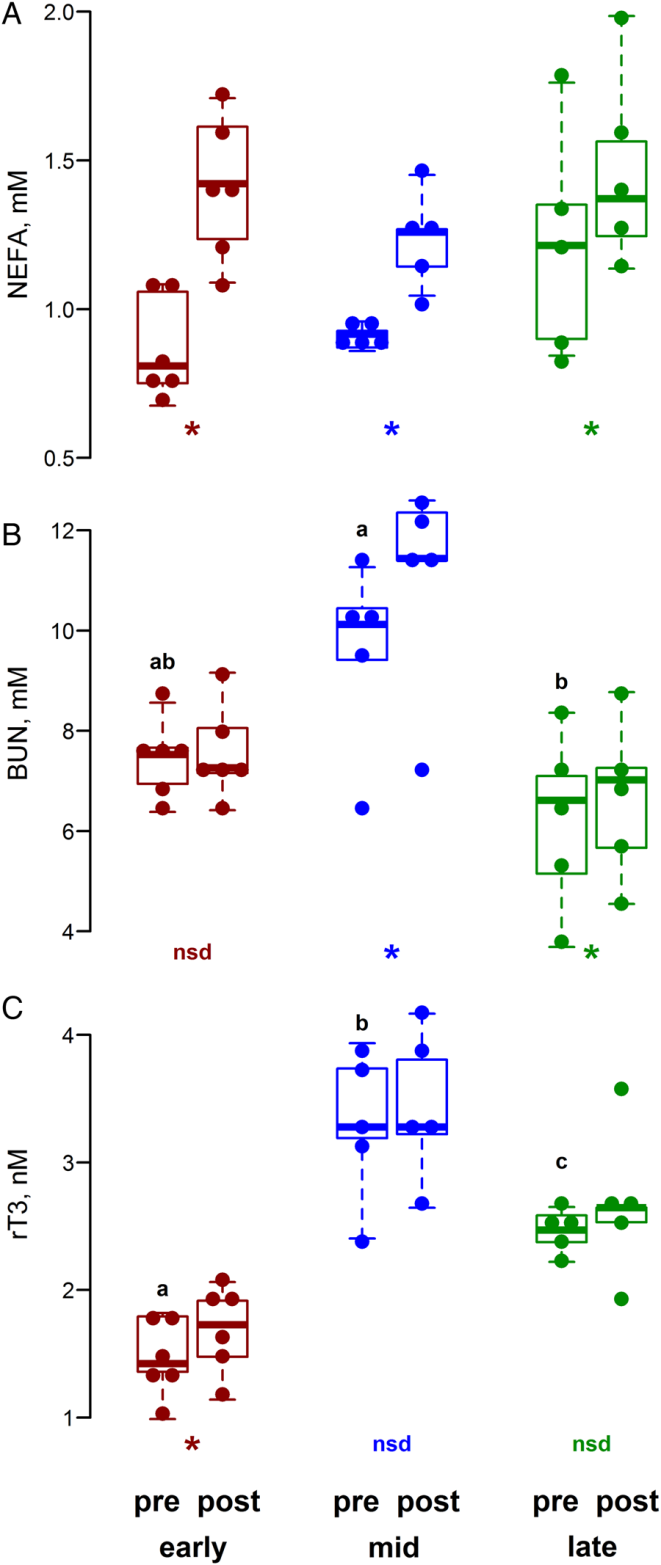
Circulating concentrations of non-esterified fatty acids (NEFA; **A**), blood urea nitrogen (BUN; **B**) and reverse triiodothyronine (rT_3_; **C**) before and 2.5 h after the administration of exogenous ACTH (pre and post, respectively) at the beginning, middle and end of the moulting period (early, mid and late, respectively). Coloured asterisks along the *x*-axis indicate a significant difference between the pre and post samples (Student's paired *t* test, *P* < 0.05); ‘nsd’ indicates no significant difference detected. Letters above the pre groups indicate differences in the initial concentrations among moulting stages (early, mid and late; ANOVA followed by Tukey's HSD test, *P* < 0.05).

We measured the responses of NEFA, BUN and rT_3_ in initial and final samples (time zero ‘pre’ samples and 150 min after exACTH administration ‘post’ samples; Fig. 6). The NEFA levels increased in response to exACTH administration in all three study groups (Student's paired *t* = 9.0, 4.2 and 6.0 in early, mid and late groups, respectively; *P* < 0.05), and the magnitude of response decreased across moulting (based on the proportional increase in NEFA between pre and post samples; *F*_2,13_ = 6.2, *P* = 0.013; Tukey's *P* < 0.05). Blood urea nitrogen showed a more variable response; there was no detectable difference in BUN concentration following exACTH administration early in the moult (*P* > 0.1), a significant increase in the middle of the moult (Student's paired *t* = 4.1, *P* < 0.05) and a small but statistically significant increase at the end of moulting (Student's paired *t* = 4.2, *P* < 0.05). There was not a strong response of rT_3_ to exACTH; there was a small but statistically significant increase early in moulting (Student's paired *t* = 4.3, *P* < 0.01) and no detectable change in the middle or end of the moult (*P* > 0.1; Fig. 6C).

**Figure 7:**
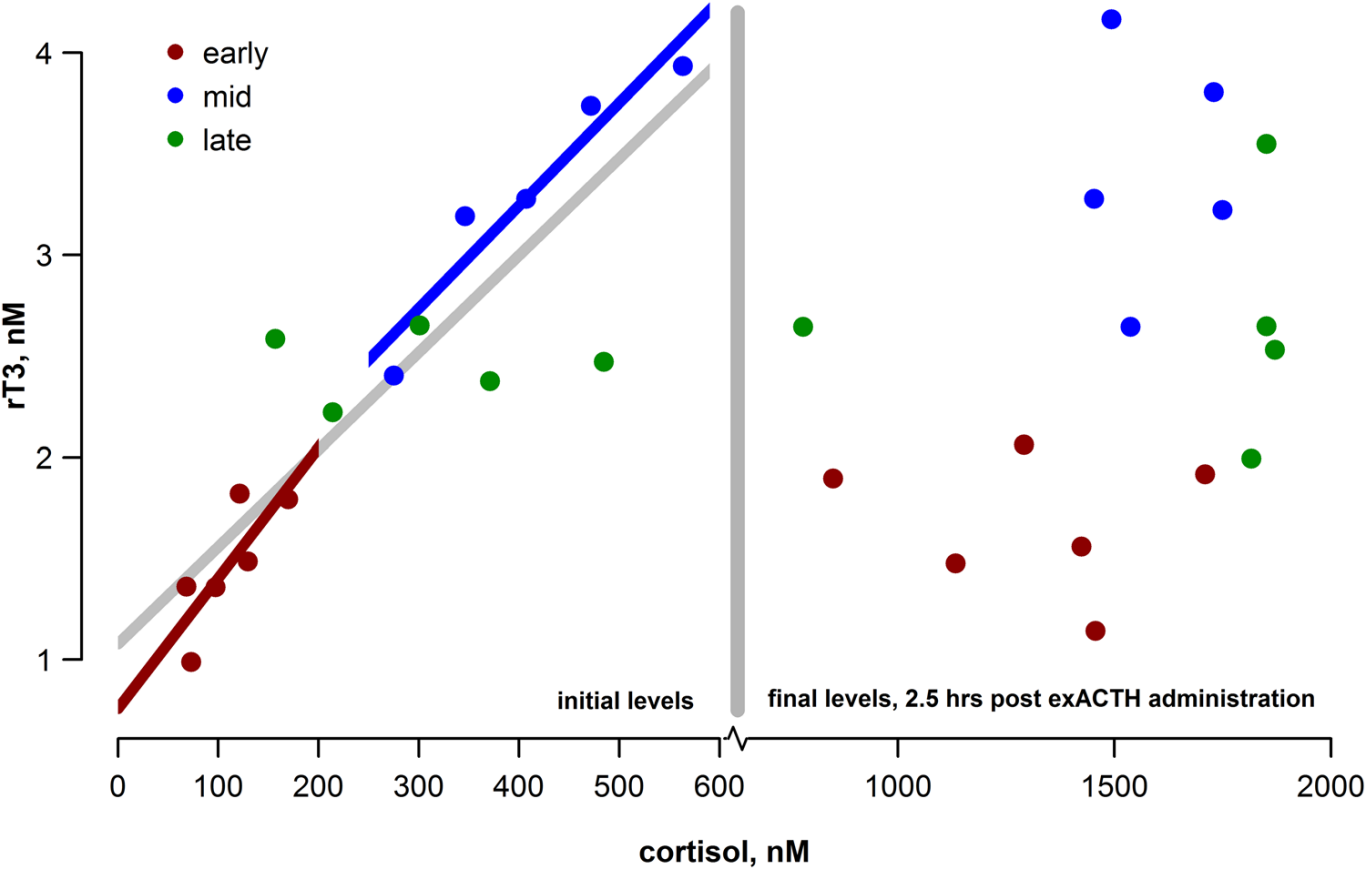
Initial values of reverse triiodothyronine (rT_3_) and cortisol were tightly correlated (left panel; *F*_1,10_ = 210.5, *P* < 0.001; linear fit shown by the grey line) and the slopes varied by study group (cortisol × state, *F*_2,10_ = 10.3, *P* < 0.01). This association disappeared during an acute stress response (right panel; *P* > 0.1). Note that the *x*-axis scale differs between the two panels.

To assess the relationship between rT_3_ and cortisol, we ran a general linear model with moulting state as a cofactor. There was a close association between initial rT_3_ and cortisol concentrations (cortisol, *F*_1,10_ = 210.5, *P* < 0.0001; Fig. 7), and this relationship varied among study groups (cortisol × state, *F*_2,10_ = 10.3, *P* < 0.01). At the end of moulting, rT_3_ did not vary with cortisol (*P* > 0.9), whereas there were close ­associations during the early and mid-moulting stages (early, *r*^2^ = 0.54, *P* = 0.058; mid, *r*^2^ = 0.88, *P* = 0.01). This association between initial rT_3_ and cortisol concentrations was no longer present 150 min after exACTH administration (*P* > 0.1; Fig. 7).

## Discussion

Stimulating the HPA axis with exACTH caused a co-ordinated hormonal and metabolic response in juvenile northern elephant seals. Baseline cortisol and aldosterone concentrations varied 4- to 6-fold during moulting. Despite the fluctuations in the initial corticosteroid concentrations, study animals responded in a similar manner to exACTH administration and reacted with comparable corticosteroid secretion relative to baseline concentrations. Most studies administer 0.5–2.0 U/kg of exACTH (e.g. Wasser *et al.*, 2000; Lien and Huang, 2008). Based on preliminary trials, we used a lower dose of 0.25 U/kg, which resulted in a 3- to 12-fold increase in both cortisol and aldosterone. The response to exACTH administration resulted in significant state-dependent downstream effects on glucose metabolism, lipid availability and protein catabolism, indicated by changes in circulating concentrations of glucose, lactate, NEFA and BUN, respectively.

Baseline concentrations of both cortisol and aldosterone varied substantially and followed similar patterns throughout moulting, with the highest hormone concentrations in the ­middle of the moult (Table 1). The concentration of ACTH, however, remained relatively consistent throughout moulting, suggesting an allostatic adjustment altering corticosteroid levels during this life-history stage. Some hormone and metabolite concentrations varied among the study groups following the exACTH administration (e.g. peak cortisol concentrations differed between groups 2.5 h post-administration), but these differences were largely driven by initial conditions. The total response, best measured as AUC values, was not detectably different among study groups for any response variable (e.g. see Fig. 2 insets for cortisol, aldosterone and ACTH). These data suggest that seals maintained the ability to respond to additional stressors despite changes in baseline stress ­hormone concentrations. Both cortisol and aldosterone increased immediately following exACTH administration. Despite this increase, elephant seals did not display a significant decrease in ACTH for at least 2 h after exACTH administration (Fig. 2), suggesting delayed negative feedback on the HPA axis.

Cortisol and aldosterone showed similar responses following the administration of exACTH; concentrations increased rapidly for ~60 min and continued to increase for the duration of the 2.5 h sampling period, but at a reduced rate (Fig. 2A and B). The response of cortisol was similar among study groups; average peak cortisol concentrations varied only between 1300 and 1600 nm. There were no detectable differences in total aldosterone release among study groups (inset in Fig. 2B). The greater variability in baseline aldosterone concentrations probably influenced the differences apparent among study groups in the absolute aldosterone concentrations following exACTH administration. Rehabilitated (Gulland *et al.*, 1999) and captive (Keogh, 2011) harbour seals (*Phoca vitulina*) showed similar patterns following exACTH administration, with sharp increases in aldosterone and cortisol for ~1 h, a plateau, followed by decreased concentrations after ~90 min and 2.5 h, respectively. These studies were conducted in the laboratory rather than field settings and administered an exACTH dose of 0.5 U/kg (Keogh, 2011) and 1.0 U/kg (Gulland *et al*., 1999), whereas we used a lower mass-specific dose of 0.25 U/kg. Despite this lower dose, cortisol concentrations in elephant seals increased to over 1000 nm in all study groups, nearly twice what was observed in harbour seals. These data suggest that, despite the apparently resilient nature of elephant seals, their HPA axis is quite sensitive to perturbation.

The responses of cortisol and aldosterone were tightly ­correlated (Fig. 3), further suggesting they are regulated in a co-ordinated manner by ACTH. These hormones share similar synthetic pathways; cortisol is produced by 11β-hydrolase (coded for by gene *CYP11B1* and regulated by ACTH), whereas aldosterone is produced by aldosterone synthase (coded for by a similar gene, *CYP11B2*, and regulated by the renin–­angiotensin–aldosterone system, in humans). An unequal crossover event between these genes can result in a gene variant with a *CYP11B1* regulatory element and *CYP11B2* catalytic sequence, i.e. regulated by ACTH but coding for aldosterone synthase activity, resulting in glucocorticoid-suppressible hyperaldosteronism (Pascoe *et al.*, 1992; White and Pascoe, 1992). Cortisol and aldosterone seem to be associated more closely in phocid seals than commonly reported in terrestrial mammals. Aldosterone is typically ­regulated by the ­renin–angiotensin–aldosterone system in mammals. This system is activated by reduced renal blood flow and results in production of angiotensin I, which is converted to angiotensin II by angiotensin-­converting enzyme (found primarily in the lungs) and subsequently stimulates aldosterone secretion (Porterfield and White, 2007). In a diving mammal, however, renal blood flow varies (Zapol *et al.*, 1979) and the lungs are often not perfused (Ponganis, 2011), potentially limiting angiotensin II production. Thus, the regulation of aldosterone secretion by ACTH may be advantageous in a diving mammal. This potential explanation is purely speculative, but the mechanism of the association between ACTH and co-ordinated cortisol and aldosterone production deserves further investigation.

We found a close association between cortisol and rT_3_ in the baseline state but not during acute stress (Fig. 7). Stress typically decreases circulating thyroid hormones, triiodothyronine (T_3_) and thyroxine (T_4_), reducing whole-animal energy use, during food limitation, for example (Servatius *et al.*, 2000; Helmreich *et al.*, 2005). The reduction in T_3_ concentration may be facilitated by increased conversion of T_4_ to rT_3_ by an inner-ring deiodinase, primarily D3 (Bianco and Kim, 2006); rT_3_ binds with T_3_ receptors but has no biological activity, thus acting as a T_3_ blocker, further enhancing metabolic suppression. There is some evidence that rT_3_ concentrations increase during GC treatment in humans (Chopra *et al.*, 1975). The present study suggests that rT_3_ may track cortisol concentrations over longer time periods (more than several hours). Acute stress events, such as animal handling, may not affect rT_3_ concentrations. Thus, rT_3_ may provide a marker of stress in free-ranging animals, because it may be disassociated from proximate handling effects, but further study is required to describe the relationship between stress, GCs and the timing and magnitude of the rT_3_ response.

In addition to the hormonal changes that occurred following exACTH administration, we evaluated several downstream effects on metabolism (Breuner *et al.*, 2013). Many metabolic features were influenced by the stress response, including lipolysis, protein, and carbohydrate metabolism. We use metabolite values here as indices of whole-animal metabolism; NEFA, BUN, glucose and lactate concentrations as indicators of lipolysis, protein and carbohydrate metabolism, respectively. However, we urge caution when using static metabolite values to infer changes in metabolic pathways; sometimes these are correlated, but often they are not (e.g. see Houser *et al*., 2007).

Usually, the primary metabolic role of stress-induced GC release is to increase glucose availability in the circulation (Sapolsky *et al.*, 2000). In the present study, however, NEFA was the metabolite most influenced by exACTH administration, increasing by 20–60% compared with initial levels, far greater than the 15–20% increase observed in glucose concentration. These increased NEFA concentrations may result either from increased lipolysis from abundant adipose stores in this species or from reduced fatty acid re-esterification to triglyceride. The increase in NEFA occurred despite already high baseline rates of lipolysis typical of phocid seals (>10 μmol/kg/min; Crocker *et al.*, 2014). The rate of fatty acid re-esterification is also high in elephant seals (Houser *et al.*, 2007), and the increase in circulating NEFA may result from decreased re-esterification induced by GCs rather than increased lipid mobilization (Jeanrenaud, 1967). Despite the lack of any detectable difference among initial NEFA concentrations, the magnitude of response, relative to baseline concentrations, declined with the progression of moulting. The action of GCs on lipolysis is modulated by dehydrogenases that convert GCs between active (e.g. cortisol) and inactive forms (e.g. cortisone; Peckett *et al.*, 2011). Adipose tissue contains high levels of the activating dehydrogenase that can lead to substantially higher concentrations of GCs at target tissues than those present in the circulation (Masuzaki *et al.*, 2001). Local regulation within adipose tissue may therefore result in high responsiveness to GCs and modulate the response with changing conditions.

The stress response had a variable effect on protein catabolism during moulting, indicated by alterations in BUN ­concentration. The considerable baseline variability in BUN concentrations suggests that the rate of protein catabolism or urea clearance changes during moulting. High BUN concentrations in the middle of the moult suggest that this period may be associated with skin synthesis, which requires amino acid mobilization from lean tissue. In response to exACTH administration, BUN increased substantially in the middle of the moult, when initial concentrations were already at their highest levels, but the magnitude of BUN increase was much lower at the end of moult and was unaffected early in the moult (Fig. 6B). Peak cortisol concentrations reached similar levels at the middle and end of moulting, but the response by BUN was vastly different, increasing only marginally at the end of moulting. This difference in BUN response following exACTH administration may indicate that the sensitivity of lean tissue to cortisol varies while moulting. Northern elephant seals typically show very low rates of protein catabolism during fasting (Crocker *et al.*, 1998; Houser and Costa, 2001). The apparent increased influence of cortisol on protein catabolism at this particular stage might suggest that active moulting may be one of the few times when protein mobilization is advantageous in this species.

Carbohydrate metabolism was significantly influenced by exACTH and the resultant cortisol release. Cortisol typically increases glucose availability by promoting gluconeogenesis in the liver and amino acid release from lean tissue to provide gluconeogenic substrates (Kraus-Friedmann, 1984). Glucose concentrations increased in each study group; interestingly, the most rapid increase occurred early in moulting, when there was no detectable increase in BUN, an index of protein catabolism. In contrast, the most delayed increase in glucose concentration was in the middle of the moult, during the largest BUN increase. Together, these data do not support a close association between cortisol release, protein catabolism and gluconeogenesis in this species. Rather, the increase in glucose was accompanied by a parallel reduction in lactate. Following exACTH administration, these metabolites displayed similar responses in the timing of their change compared with baseline, requiring 30–45, 90 and 120 min in early, mid, and late moulting, respectively (Fig. 4), and their concentrations were inversely correlated during the stress response (Fig. 5).

One typical metabolic consequence of the stress response is that cortisol release stimulates protein catabolism and the release of amino acids into the circulation, providing substrates for gluconeogenesis and increasing glucose availability for immediate energy needs (Sapolsky *et al.*, 2000). This protein catabolism can result in lean tissue degradation during chronic or repeated stress events. Animals adapted to prolonged fasts, such as many marine mammals, including elephant seals, have very low rates of protein degradation (Crocker *et al.*, 1998; Houser and Costa, 2001; Verrier *et al.*, 2009) to maintain organ function during fasts associated with breeding, lactation, development and moulting (Champagne *et al.*, 2012a). Strong increases in glucose demands are also evident during extensive tissue repair and synthesis (Williams and Barbul, 2003). Changing hepatic responsiveness to glucocorticoids across moulting may reflect conflicting metabolic demands for new pelage synthesis and the need to spare mobilized protein. Adaptations to fasting may consequently protect lean tissue from degradation during stress. The substrates required for glucose production may be supplied from other intermediates, such as lactate (Champagne *et al.*, 2006, 2012c; Tavoni *et al.*, 2013). The close association between lactate and glucose (Fig. 5) and the lack of a strong response in BUN concentrations are consistent with this hypothesis (Fig. 6).

The response to exACTH administration was recently reported in adult male northern elephant seals during different life-history stages, namely breeding and mid-moulting (Ensminger *et al.*, 2014). A lower mass-specific dose was used in the much larger adults (0.15 U/kg), but several similarities were observed between their study and the present study. Cortisol increased in both age classes (juveniles and adult males), while ACTH did not show strong suppression, suggesting impaired or delayed negative feedback of the HPA axis in this species. Likewise, aldosterone increased in all study groups, further indicating that aldosterone is, at least in part, regulated by ACTH independently of the renin–angiotensin–aldosterone system. There was no detectable influence of the transient increase in aldosterone on circulating sodium or potassium concentrations in adult males (these were not measured in the present study). Adult males also did not show a rapid increase in rT_3_ following exACTH administration; rT_3_ increased only 48 h following the exACTH administration and then only in a single study group (early in the breeding season). Overall, this suggests a delayed influence of increased cortisol on rT_3_ concentrations that may be useful in distinguishing between transient and sustained stressors. Glucose and lactate did not respond as strongly to exACTH administration in adult males, but the total responses (measured by AUC) were similar in the two age classes. The consistent relationship between glucose and lactate across age classes and life-history stages supports the hypothesis that lactate is the primary gluconeogenic precursor in elephant seals and may be a common metabolic feature of fasting in marine mammals (Champagne *et al.*, 2012a,c). Circulating NEFA increased following exACTH administration during moulting in both juveniles and adults; breeding adults, however, did not show a detectable increase in NEFA. Protein catabolism is generally low in fasting elephant seals. In the present study, exACTH administration substantially increased BUN concentrations in only one of the three study groups, mid-moulting, and Ensminger *et al.* (2014) did not detect an influence on BUN in adult males among any study group, during breeding or moulting. This may suggest that the resultant aldosterone release following exACTH administration influences rates of renal filtration and urea clearance or that there is a limited period during moulting when protein catabolism is sensitive to cortisol release in elephant seals.

We simulated a stress event by administering exACTH to a model marine mammal species, the northern elephant seal, and evaluated the resulting acute stress response. We found that the HPA axis remained sensitive to stimulation, despite the substantial variation in baseline GC levels that occurred during moulting, suggesting that seals are resilient to the normal variation in GC levels that occur during their normal life histories. Both cortisol and aldosterone increased concurrently during acute stress. Aldosterone is not typically considered part of the stress response in most mammals, but numerous studies have now confirmed its responsiveness to stressors in a variety of marine mammals (Thomson and Geraci, 1986; St Aubin and Dierauf, 2001; Houser *et al.*, 2011). The role played by aldosterone in an adaptive stress response, if any, is currently unknown. Lipid and carbohydrate metabolism were altered during stress, as shown by changes in circulating concentrations of NEFA, glucose and lactate. Circulating BUN concentrations, however, showed far smaller changes, suggesting a reduced influence of stress on protein catabolism in this species. In order to gain a better understanding of the consequences of stress on metabolism and energy use, studies combining stress with additional monitoring techniques (e.g. respirometry or metabolic tracers) will be needed. This work provides further support that baseline circulating GC levels alone are not reliable indicators of stress in free-ranging animals; the natural variation during season and life-history stage must be appreciated before using GC measurements as indices of stress.

## Funding

This work was performed under National Marine Fisheries Service permit 14636, supported by the Office of Naval Research (grant no. N000141110434) and approved by the Sonoma State University Institutional Animal Care and Use Committee.
